# Crystal structure of 4′-bromo-2,5-dihy­droxy-2′,5′-dimeth­oxy-[1,1′-biphen­yl]-3,4-dicarbo­nitrile

**DOI:** 10.1107/S2056989016005715

**Published:** 2016-04-12

**Authors:** Joseph E. Meany, Steven P. Kelley, Robin D. Rogers, Stephen A. Woski

**Affiliations:** a Department of Chemistry, The University of Alabama, Box 870336, Tuscaloosa, AL 35487-0336, USA; b Department of Chemistry, McGill University, 801 Sherbrooke St. West, Montreal, Quebec, H3A 0B8, Canada

**Keywords:** crystal structure, hemibi­quinone, mol­ecular rectifier

## Abstract

In the crystal of the title substituted hemibi­quinone derivative, C_16_H_11_BrN_2_O_4_ or [BrHBQH_2_(CN)_2_], the substituted benzene rings are rotated about the central C—C bond, forming a dihedral angle of 53.59 (7)°. The ring systems inter­act through an intra­molecular O—H⋯O_meth­oxy_ hydrogen bond, which induces a geometry quite different from those in previously reported hemibi­quinone structures. In the crystal, the mol­ecules associate through an inter­molecular O—H⋯N_nitrile_ hydrogen bond, forming chains which extend along [100] and are inter­linked through very weak C—H⋯N hydrogen bonds, giving a overall two-dimensional structure lying parallel to (010).

## Abstract 2

In the crystal of the title substituted hemibi­quinone derivative, the ring systems inter­act through an intra­molecular O—H⋯O_meth­oxy_ hydrogen bond, which induces a geometry quite different from those in previously reported hemibi­quinone structures. The mol­ecules associate through an inter­molecular O—H⋯N_nitrile_ hydrogen bond and are inter­linked through very weak C—H⋯N hydrogen bonds.

## Chemical context   

Recently, a new class of mol­ecules (hemibi­quinones, HBQs) has been reported as potential mol­ecular rectifiers (Meany *et al.*, 2015 ▸). Biphenyl derivatives have garnered great attention as conductors of electricity (Venkataraman *et al.*, 2006 ▸). The symmetric nature of the biphenyl and polyphenyl derivatives studied so far allows for reasonable conduction through the π orbitals. Biphenyl derivatives with one electron-rich and one electron-deficient ring may be able to preferentially bias the direction of electron flow through the mol­ecule, thus acting as a mol­ecular diode. The donor–bridge–acceptor model has long been accepted as a basis for the design of mol­ecular rectifiers (Aviram & Ratner, 1974 ▸). The asymmetric biphenyl structure should allow for conductivity through each of the rings, while the dihedral angle between the two rings decreases orbital overlap and allows for partial isolation of the electron-rich donor and electron-poor acceptor moieties. The efficiency of conduction through a given mol­ecule can be tuned depending on the torsion angle between the two rings.
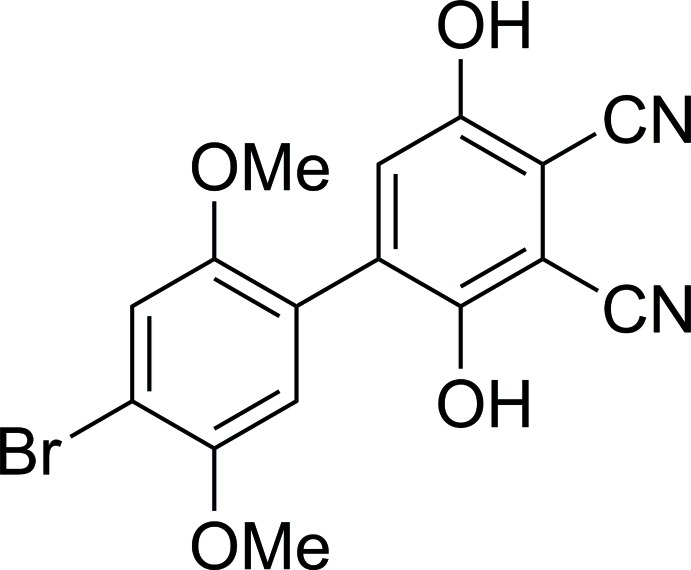


As one of the series of mol­ecules made for testing rectification through HBQs, the title compound, C_16_H_11_BrN_2_O_4_, [BrHBQH_2_(CN)_2_], (I) has been isolated as an inter­mediate in the preparation of an HBQ derivative which can self-assemble on a gold surface. We have developed a selective synthesis for this reduced hemibi­quinone derivative that is scalable to gram qu­anti­ties. Mol­ecule (I) is predicted not to act as a mol­ecular diode itself because both rings act as donor moieties. The oxidation of the hydro­quinone ring of (I) would produce a potential rectifier.

Di­cyano-functionalized hydro­quinones are known for their ability to form hydrogen-bonded networks (Reddy *et al.*, 1996 ▸) and charge-transfer complexes (Bock *et al.*, 1996 ▸), sometimes both at once (Ghorai & Mani, 2014 ▸). They have also been used as rigid ligands in coordination polymers (Kuroda-Sowa *et al.*, 1997 ▸). However, there are no crystal structures in which a di­cyano-functionalized hydro­quinone moiety has been appended to another aromatic ring. The present study affords an opportunity to investigate the mutual effects of these two functionalized ring systems on both the geometry of the mol­ecule and its inter­molecular inter­actions.

## Structural commentary   

**Figure 1:**
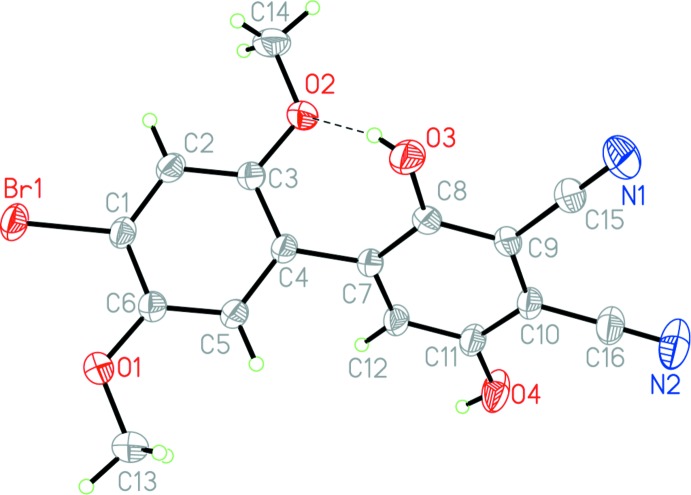
The mol­ecular structure of (I), showing the atom-numbering scheme. Displacement ellipsoids are displayed at the 50% probability level. The intra­molecular O3—H⋯O2 hydrogen bond is shown as a dashed line.

In the title compound (Fig. 1 ▸), the benzene rings are twisted out of a common plane, forming a dihedral angle of 53.59 (7)°, which appears to optimize the 2.7576 (18) Å O3—H⋯O2 intra­molecular hydrogen bond (Table 1 ▸). The rings are essentially planar although the O3—H group, which participates in the intra­molecular hydrogen bond, is displaced slightly out of the plane. Also, the rings are not co-axial with the C4—C7 bond that bridges them. This can be seen in torsion angles involving three carbon atoms from one ring and the bridging carbon atom from the other, which deviate from linearity by *ca* 5° [C2—C3—C4—C7 = 173.88 (14)°, C6—C5—C4—C7 = −175.45 (14)°, C4—C7—C8—C9 = 174.94 (13)°, C4—C7—C12—C11 = −175.62 (13)°]. This bending of the mol­ecule about its long axis may also be due to hydrogen bonding as it causes the meth­oxy group to approach the OH group more closely. The aromatic C—C bonds of both rings have a narrow range of distances [from 1.387 (2) to 1.412 (2) Å]. The C—C, C—O, C—N, and C N distances for the mol­ecule are similar to the corresponding distances in 2,3,5,6-tetra­cyano­hydro­quinone (Bock *et al.*, 1993 ▸). The C—C bond distances around the bromo­dimeth­oxy­benzene ring are close to those in the other hemibi­quinone mol­ecules containing this ring (Meany *et al.*, 2015 ▸, 2016 ▸). The C9—C10 bond in (I) [1.408 (2) Å] is longer than the corresponding C1—C6 bond in BrHBQBr (1.334 Å; Meany *et al.*, 2015 ▸). The stronger polarization of (I) relative to the starting material should weaken the bond through repulsive effects. The Br1—C1 bond is slightly shorter in (I) [1.885 (1) Å] compared to the starting material [1.898 (4) Å] as well, also suggesting decreased electron density on the di­meth­oxy­benzene ring due to increased polarization. The calculated dipole (B3LYP-DGDZVP) of BrHBQBr is only 1.33 D, compared to 6.17 D for (I).

**Figure 2:**
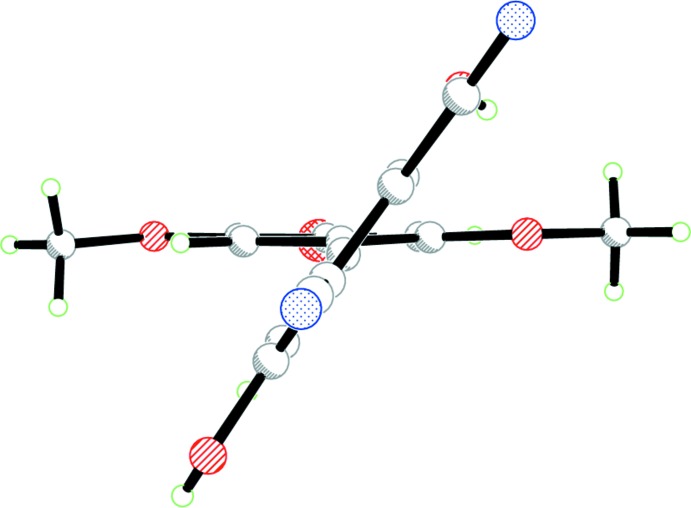
Ball-and-stick plot of (I), viewed down the C4—C7 bond.

As in the other reported hemibi­quinone mol­ecules (Meany *et al.*, 2015 ▸), we seek to use and compare the inter-ring torsion angles in the crystals as a guide compared to gas-phase calculated values. The intra­molecular hydrogen bond from the C8 phenol to the O2 meth­oxy group causes a greater torsion angle than that in the starting HBQ (Meany *et al.*, 2015 ▸). In (I), the C5—C4—C7—C8 torsion angle is −126.5 (2)°, compared to −110.9 (5)° in HBQ. DFT (B3LYP-DGDZVP) calculations performed on the target mol­ecule in the gas phase predict an angle of 48.85°. This significant discrepancy is due to packing inter­actions in the solid phase as well as the additional hydrogen bond. The hydrogen bond is indicated in Fig. 1 ▸, while the relative orientations of the rings can be seen in Fig. 2 ▸.

The O3—H⋯O2 intra­molecular hydrogen bond points toward the non-bonded electrons on O2 with a total bond angle of 152 (3)°. As a result of the influence of other short contacts and supra­molecular inter­actions (see below), the phenolic C—O—H bond angles deviate when compared to the meth­oxy C—O—C bond angles: C8—O3—H is 108 (2)°, C11—O4—H is 112.3 (2)°, C3—O2—C14 is 117.9 (1)°, and C6—O1—C13 is 117.2 (1)°. As in other structures, the meth­oxy groups are aligned mostly in-plane with the benzene ring, C5— C6—O1—C13 being bent out of plane by −4.5 (2)° and C2—C3—O2—C14 bent out of plane by −1.3 (2)°. The C12—C11—O4—H phenol group is also nearly planar, being bent out of plane by 1.3°. However, the hydrogen-bonded phenol is unsurprisingly bent out of plane, C7—C8—O3—H = 38 (2)°. The meth­oxy methyl groups point away from the sterically restricting groups *ortho* to these positions.

## Supra­molecular features   

**Figure 3:**
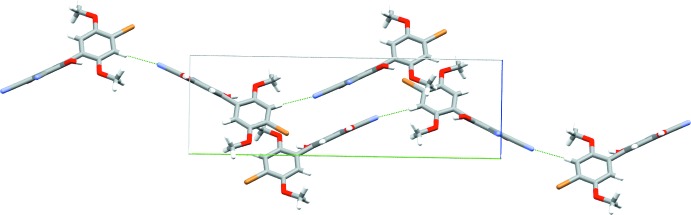
Short (less than the sum of the van der Waals radii) contact environment around [BrHBQH_2_(CN)_2_]. Dashed green lines indicate short contacts. Axes are color coded: red = *a* axis, green = *b* axis and blue = *c* axis.

**Figure 4:**
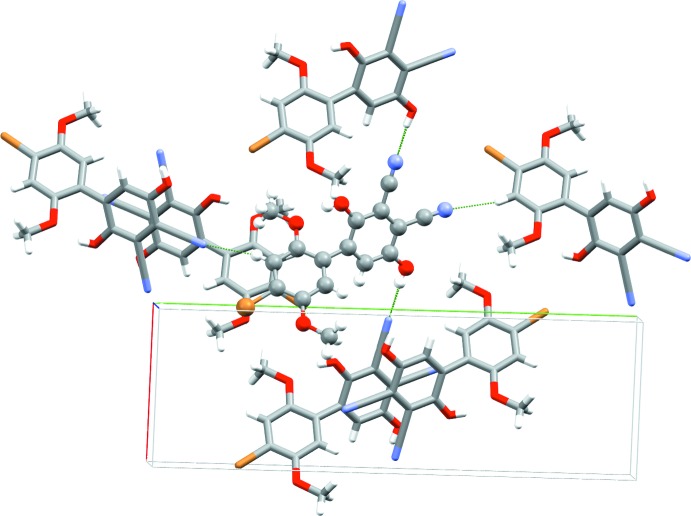
Hydrogen-bonded sheets along *ab*. Dashed green lines indicate short contacts. Axes are color coded: red = *a* axis, green = *b* axis and blue = *c* axis.

Each mol­ecule makes short (less than the sum of the van der Waals radii) contacts to six neighboring mol­ecules (Fig. 3 ▸). As in previously reported HBQ structures, rings of like identity are all aligned in parallel planes. All short contacts are associated with Lewis acid–base inter­actions of some kind, and for each inter­action there is one neighboring mol­ecule that acts as a donor and second that acts as an acceptor. Two central mol­ecules in the unit cell stack anti­parallel to one another, the quinone rings shifted off-center from one another in the *a*-axis direction. Both nitrile groups are involved in inter­molecular hydrogen-bonding inter­actions, the first one (O4—H⋯N1) strong, the second one (C2—H⋯N2) weaker but still highly directional. For details, see Table 1 ▸. These inter­actions link mol­ecules along the crystallographic *a*- and *b*-axis directions, respectively, forming sheets parallel to (010) (Fig. 4 ▸). The quinone rings are aligned parallel to the *bc* plane diagonal.

**Figure 5:**
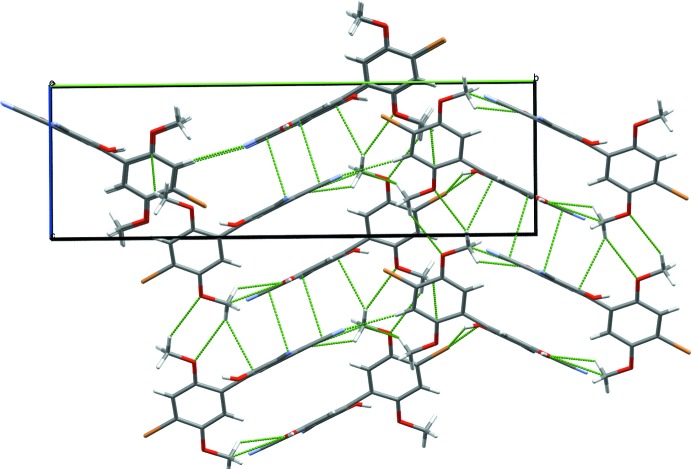
Unit-cell packing of (I), viewed along the *a* axis. Short contacts show the long ring stacking along the *c* axis.

The remaining two mol­ecules in the unit cell are oriented orthogonally to the central mol­ecules. These mol­ecules are anti­parallel to each other, where the di­meth­oxy­benzene rings stack with those of the central pair. Slightly repulsive π-inter­actions between mol­ecules along *b* and stacking along *c* can be seen in Fig. 5 ▸. Inter­centroid distances for the rings are longer than expected for close π–π inter­actions at 4.107 (1) Å. However, since the rings are slightly offset from one another, this is not the correct centroid to use. Instead, a close 3.598 (1) Å π-inter­action between two inter­molecular C9—C10 centroids exists. A centroid calculated for the C7—C8—C9—C11—C12 ring sits 3.574 (1) Å from a centroid for N1—C15—C9—C10—C16—N2, which may be explained by the electron-donating character of the hydro­quinone as compared to the di­nitrile substituents. The planes of the di­meth­oxy­benzene rings are oriented parallel to the short diagonal of the *ac* plane.

## Synthesis and crystallization   

2-Bromo-5-(4-bromo-2,5-di­meth­oxy­phen­yl)cyclo­hexa-2,5-diene-1,4-dione, BrHBQBr, (0.300 g, 0.744 mmol) was dissolved in 350 mL of aceto­nitrile. In a separate beaker, potassium cyanide (0.124 g, 1.90 mmol) was dissolved in 50 mL of H_2_O. Upon pouring the aqueous solution into the organic solution, the mixture immediately changed from a vibrant red to a deep purple. After stirring for 1 h, 50 µL of concentrated HCl solution was added, changing the color of the mixture from purple to bright orange. The mixture was diluted with 50 mL of water and the aceto­nitrile was removed by rotary evaporation. A tan powder precipitated, which was recovered by filtration and washed with water to yield the crude product. This material was recrystallized from acetone giving 0.196 g (70.4%) of pure material as yellow–orange prisms. ^1^H NMR (360 MHz, *d*_6_-acetone) δ = 10.02 (*s*, 1H, ArOH), 8.75 (*s*, 1H, ArOH), 7.34 (*s*, 1H, ArH), 7.24 (*s*, 1H, ArH), 7.05 (*s*, 1H, ArH), 3.88 (*s*, 3H, OCH_3_), 3.82 (*s*, 3H, OCH_3_).

## Refinement   

Crystal data, data collection and structure refinement details are summarized in Table 2 ▸. Hydroxyl hydrogen atoms were located from the difference map and their coordinates were refined while the thermal parameters were constrained to ride on the carrier atom with *U*_iso_ = 1.5*U*_eq_(O). Hydrogen atoms bonded to carbon were placed in calculated positions with C—H = 0.93 Å (aromatic) or 0.96 Å (meth­yl) and their coordin­ates and thermal parameters were constrained to ride on the carrier atom, with *U*_iso_ = 1.5*U*_eq_(aromatic C) or 1.5*U*_eq_(methyl C).

## Supplementary Material

Crystal structure: contains datablock(s) I. DOI: 10.1107/S2056989016005715/zs2361sup1.cif

Structure factors: contains datablock(s) I. DOI: 10.1107/S2056989016005715/zs2361Isup2.hkl

Supporting information file. DOI: 10.1107/S2056989016005715/zs2361Isup3.cml

CCDC reference: 1472611

Additional supporting information:  crystallographic information; 3D view; checkCIF report

## Figures and Tables

**Table 1:** Hydrogen-bond geometry (Å, °)

*D*—H⋯*A*	*D*—H	H⋯*A*	*D*⋯*A*	*D*—H⋯*A*
O3—H3*A*⋯O2	0.72 (3)	2.11 (3)	2.7576 (18)	152 (3)
O4—H4*A*⋯N1^i^	0.79 (2)	2.03 (2)	2.8189 (18)	172 (2)
C2—H2*A*⋯N2^ii^	0.93	2.72	3.638 (2)	168

Symmetry codes: (i) 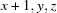; (ii) 
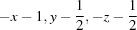
.

**Table 2:** Experimental details

Crystal data	
Chemical formula	C_16_H_11_BrN_2_O_4_
*M*_r_	375.18
Crystal system, space group	Monoclinic, *P*2_1_/*c*
Temperature (K)	296
*a*, *b*, *c* (Å)	8.4726 (3), 23.7748 (8), 8.0833 (3)
β (°)	111.6985 (17)
*V* (Å^3^)	1512.88 (9)
*Z*	4
Radiation type	Mo *K*α
μ (mm^−1^)	2.74
Crystal size (mm)	0.35 × 0.20 × 0.09

Data collection	
Diffractometer	Bruker APEXII CCD
Absorption correction	Multi-scan (*SADABS*; Bruker, 2010 ▸)
*T*_min_, *T*_max_	0.428, 0.747
No. of measured, independent and observed [*I* > 2σ(*I*)] reflections	65083, 6269, 4739
*R*_int_	0.037
(sin θ/λ)_max_ (Å^−1^)	0.796

Refinement	
*R*[*F* ^2^ > 2σ(*F* ^2^)], *wR*(*F* ^2^), *S*	0.035, 0.089, 1.03
No. of reflections	6269
No. of parameters	216
H-atom treatment	H atoms treated by a mixture of independent and constrained refinement
Δρ_max_, Δρ_min_ (e Å^−3^)	0.59, −0.26

Computer programs: *APEX2* and *SAINT* (Bruker, 2010 ▸), *SHELXS97* and *SHELXTL* (Sheldrick 2008 ▸) and *SHELXL2014* (Sheldrick, 2015 ▸).

## supplementary crystallographic information

### Crystal data

**Table:**

C_16_H_11_BrN_2_O_4_	*F*(000) = 752
*M**_r_* = 375.18	*D*_x_ = 1.647 Mg m^−^^3^
Monoclinic, *P*2_1_/*c*	Mo *K*α radiation, λ = 0.71073 Å
*a* = 8.4726 (3) Å	Cell parameters from 9370 reflections
*b* = 23.7748 (8) Å	θ = 2.6–29.9°
*c* = 8.0833 (3) Å	µ = 2.74 mm^−^^1^
β = 111.6985 (17)°	*T* = 296 K
*V* = 1512.88 (9) Å^3^	Tablet, yellow-orange
*Z* = 4	0.35 × 0.20 × 0.09 mm

### Data collection

**Table:**

Bruker APEXII CCD diffractometer	4739 reflections with *I* > 2σ(*I*)
φ and ω scans	*R*_int_ = 0.037
Absorption correction: multi-scan (*SADABS*; Bruker, 2010)	θ_max_ = 34.5°, θ_min_ = 1.7°
*T*_min_ = 0.428, *T*_max_ = 0.747	*h* = −13→13
65083 measured reflections	*k* = −37→37
6269 independent reflections	*l* = −12→12

### Refinement

**Table:**

Refinement on *F*^2^	Primary atom site location: structure-invariant direct methods
Least-squares matrix: full	Secondary atom site location: difference Fourier map
*R*[*F*^2^ > 2σ(*F*^2^)] = 0.035	Hydrogen site location: mixed
*wR*(*F*^2^) = 0.089	H atoms treated by a mixture of independent and constrained refinement
*S* = 1.03	*w* = 1/[σ^2^(*F*_o_^2^) + (0.0431*P*)^2^ + 0.4609*P*] where *P* = (*F*_o_^2^ + 2*F*_c_^2^)/3
6269 reflections	(Δ/σ)_max_ = 0.001
216 parameters	Δρ_max_ = 0.59 e Å^−^^3^
0 restraints	Δρ_min_ = −0.26 e Å^−^^3^

### Special details

**Table:**

Geometry. All esds (except the esd in the dihedral angle between two l.s. planes) are estimated using the full covariance matrix. The cell esds are taken into account individually in the estimation of esds in distances, angles and torsion angles; correlations between esds in cell parameters are only used when they are defined by crystal symmetry. An approximate (isotropic) treatment of cell esds is used for estimating esds involving l.s. planes.

### Fractional atomic coordinates and isotropic or equivalent isotropic displacement parameters (Å^2^)

**Table:**

	*x*	*y*	*z*	*U*_iso_*/*U*_eq_	
Br1	0.00177 (2)	0.18718 (2)	0.27906 (3)	0.03808 (6)	
O1	0.09291 (15)	0.30658 (4)	0.37344 (17)	0.0372 (3)	
O2	−0.52102 (14)	0.29007 (5)	−0.19615 (17)	0.0400 (3)	
O3	−0.65570 (14)	0.38022 (5)	−0.07840 (19)	0.0393 (3)	
H3A	−0.639 (3)	0.3515 (11)	−0.093 (3)	0.059*	
O4	−0.25442 (16)	0.52226 (5)	−0.2911 (2)	0.0502 (4)	
H4A	−0.163 (3)	0.5103 (10)	−0.276 (3)	0.050*	
N1	−0.92239 (18)	0.49008 (7)	−0.2525 (3)	0.0524 (4)	
N2	−0.6248 (3)	0.59864 (7)	−0.3963 (3)	0.0594 (5)	
C1	−0.12247 (17)	0.25158 (6)	0.1689 (2)	0.0269 (3)	
C2	−0.27623 (17)	0.24472 (6)	0.0277 (2)	0.0290 (3)	
H2A	−0.3187	0.2089	−0.0098	0.035*	
C3	−0.36583 (17)	0.29233 (6)	−0.0569 (2)	0.0277 (3)	
C4	−0.30049 (16)	0.34595 (5)	−0.00356 (19)	0.0258 (2)	
C5	−0.14639 (17)	0.35126 (6)	0.1413 (2)	0.0276 (3)	
H5A	−0.1032	0.3870	0.1789	0.033*	
C6	−0.05661 (18)	0.30449 (6)	0.2301 (2)	0.0271 (3)	
C7	−0.38125 (16)	0.39804 (5)	−0.09918 (19)	0.0258 (2)	
C8	−0.55087 (16)	0.41297 (6)	−0.1289 (2)	0.0269 (3)	
C9	−0.61363 (16)	0.46445 (6)	−0.20684 (19)	0.0270 (3)	
C10	−0.51175 (16)	0.50206 (6)	−0.2583 (2)	0.0283 (3)	
C11	−0.34649 (17)	0.48643 (6)	−0.2341 (2)	0.0312 (3)	
C12	−0.28382 (17)	0.43499 (6)	−0.1544 (2)	0.0300 (3)	
H12A	−0.1728	0.4251	−0.1377	0.036*	
C13	0.1665 (2)	0.36036 (8)	0.4267 (3)	0.0523 (5)	
H13A	0.2725	0.3563	0.5253	0.078*	
H13B	0.0908	0.3832	0.4618	0.078*	
H13C	0.1860	0.3780	0.3291	0.078*	
C14	−0.5909 (2)	0.23599 (7)	−0.2582 (3)	0.0411 (4)	
H14A	−0.6967	0.2403	−0.3568	0.062*	
H14B	−0.6098	0.2161	−0.1638	0.062*	
H14C	−0.5131	0.2151	−0.2957	0.062*	
C15	−0.78630 (18)	0.47882 (7)	−0.2331 (2)	0.0346 (3)	
C16	−0.5754 (2)	0.55568 (7)	−0.3358 (2)	0.0368 (3)	

### Atomic displacement parameters (Å^2^)

**Table:**

	*U*^11^	*U*^22^	*U*^33^	*U*^12^	*U*^13^	*U*^23^
Br1	0.03661 (8)	0.02562 (8)	0.05028 (11)	0.00760 (5)	0.01403 (7)	0.01143 (6)
O1	0.0332 (5)	0.0295 (5)	0.0380 (6)	0.0005 (4)	0.0002 (5)	0.0032 (4)
O2	0.0303 (5)	0.0276 (5)	0.0480 (7)	−0.0015 (4)	−0.0019 (5)	−0.0029 (5)
O3	0.0287 (5)	0.0312 (5)	0.0611 (8)	−0.0015 (4)	0.0201 (5)	0.0066 (5)
O4	0.0361 (6)	0.0334 (6)	0.0905 (11)	0.0093 (5)	0.0344 (7)	0.0259 (6)
N1	0.0283 (7)	0.0508 (9)	0.0779 (12)	0.0084 (6)	0.0192 (7)	0.0051 (8)
N2	0.0674 (11)	0.0381 (8)	0.0724 (12)	0.0203 (8)	0.0255 (10)	0.0161 (8)
C1	0.0264 (6)	0.0219 (6)	0.0334 (7)	0.0039 (4)	0.0122 (5)	0.0046 (5)
C2	0.0286 (6)	0.0202 (6)	0.0380 (8)	0.0004 (4)	0.0120 (6)	0.0003 (5)
C3	0.0233 (5)	0.0233 (6)	0.0342 (7)	0.0003 (4)	0.0079 (5)	0.0003 (5)
C4	0.0238 (5)	0.0205 (5)	0.0325 (7)	0.0019 (4)	0.0098 (5)	0.0025 (5)
C5	0.0261 (6)	0.0208 (5)	0.0341 (7)	0.0007 (4)	0.0090 (5)	0.0009 (5)
C6	0.0254 (5)	0.0251 (6)	0.0296 (7)	0.0018 (5)	0.0088 (5)	0.0024 (5)
C7	0.0223 (5)	0.0206 (5)	0.0322 (7)	0.0021 (4)	0.0073 (5)	0.0005 (5)
C8	0.0211 (5)	0.0249 (6)	0.0330 (7)	−0.0002 (4)	0.0082 (5)	−0.0005 (5)
C9	0.0208 (5)	0.0251 (6)	0.0330 (7)	0.0031 (4)	0.0073 (5)	−0.0011 (5)
C10	0.0259 (6)	0.0229 (6)	0.0354 (8)	0.0052 (4)	0.0105 (5)	0.0035 (5)
C11	0.0262 (6)	0.0232 (6)	0.0462 (9)	0.0034 (5)	0.0158 (6)	0.0067 (6)
C12	0.0226 (5)	0.0243 (6)	0.0433 (8)	0.0044 (4)	0.0122 (5)	0.0060 (5)
C13	0.0467 (10)	0.0336 (8)	0.0546 (11)	−0.0069 (7)	−0.0069 (8)	−0.0022 (8)
C14	0.0353 (7)	0.0336 (8)	0.0483 (10)	−0.0086 (6)	0.0083 (7)	−0.0100 (7)
C15	0.0262 (6)	0.0302 (7)	0.0455 (9)	0.0038 (5)	0.0109 (6)	−0.0008 (6)
C16	0.0364 (8)	0.0297 (7)	0.0453 (9)	0.0088 (6)	0.0165 (7)	0.0063 (6)

### Geometric parameters (Å, º)

**Table:**

Br1—C1	1.8848 (13)	C5—C6	1.3878 (19)
O1—C6	1.3655 (18)	C5—H5A	0.9300
O1—C13	1.418 (2)	C7—C12	1.3876 (19)
O2—C3	1.3799 (17)	C7—C8	1.4120 (18)
O2—C14	1.4266 (19)	C8—C9	1.3892 (19)
O3—C8	1.3528 (17)	C9—C10	1.408 (2)
O3—H3A	0.72 (3)	C9—C15	1.4396 (19)
O4—C11	1.3464 (18)	C10—C11	1.3907 (18)
O4—H4A	0.79 (2)	C10—C16	1.434 (2)
N1—C15	1.137 (2)	C11—C12	1.3927 (19)
N2—C16	1.143 (2)	C12—H12A	0.9300
C1—C2	1.388 (2)	C13—H13A	0.9600
C1—C6	1.3909 (19)	C13—H13B	0.9600
C2—C3	1.3933 (19)	C13—H13C	0.9600
C2—H2A	0.9300	C14—H14A	0.9600
C3—C4	1.3933 (19)	C14—H14B	0.9600
C4—C5	1.4004 (19)	C14—H14C	0.9600
C4—C7	1.4860 (18)		
			
C6—O1—C13	117.15 (12)	C9—C8—C7	119.57 (12)
C3—O2—C14	117.91 (12)	C8—C9—C10	121.32 (12)
C8—O3—H3A	108 (2)	C8—C9—C15	118.27 (13)
C11—O4—H4A	112.3 (17)	C10—C9—C15	120.41 (13)
C2—C1—C6	121.99 (12)	C11—C10—C9	118.94 (12)
C2—C1—Br1	118.91 (10)	C11—C10—C16	119.68 (13)
C6—C1—Br1	119.09 (10)	C9—C10—C16	121.38 (12)
C1—C2—C3	118.92 (12)	O4—C11—C10	117.60 (13)
C1—C2—H2A	120.5	O4—C11—C12	122.95 (12)
C3—C2—H2A	120.5	C10—C11—C12	119.45 (13)
O2—C3—C4	115.96 (12)	C7—C12—C11	122.30 (12)
O2—C3—C2	123.42 (13)	C7—C12—H12A	118.8
C4—C3—C2	120.62 (12)	C11—C12—H12A	118.8
C3—C4—C5	118.83 (12)	O1—C13—H13A	109.5
C3—C4—C7	123.21 (12)	O1—C13—H13B	109.5
C5—C4—C7	117.87 (12)	H13A—C13—H13B	109.5
C6—C5—C4	121.56 (13)	O1—C13—H13C	109.5
C6—C5—H5A	119.2	H13A—C13—H13C	109.5
C4—C5—H5A	119.2	H13B—C13—H13C	109.5
O1—C6—C5	124.67 (12)	O2—C14—H14A	109.5
O1—C6—C1	117.32 (12)	O2—C14—H14B	109.5
C5—C6—C1	118.01 (13)	H14A—C14—H14B	109.5
C12—C7—C8	118.37 (12)	O2—C14—H14C	109.5
C12—C7—C4	118.74 (11)	H14A—C14—H14C	109.5
C8—C7—C4	122.82 (12)	H14B—C14—H14C	109.5
O3—C8—C9	117.39 (12)	N1—C15—C9	179.5 (2)
O3—C8—C7	122.99 (13)	N2—C16—C10	179.4 (2)
			
C13—O1—C6—C1	175.14 (15)	C4—C5—C6—O1	−179.10 (15)
C13—O1—C6—C5	−4.5 (2)	C4—C5—C6—C1	1.2 (2)
C14—O2—C3—C2	−1.3 (2)	C4—C7—C8—O3	−2.4 (2)
C14—O2—C3—C4	178.48 (15)	C4—C7—C8—C9	174.94 (13)
Br1—C1—C2—C3	−178.26 (12)	C12—C7—C8—O3	−179.24 (14)
C6—C1—C2—C3	1.0 (2)	C12—C7—C8—C9	−1.9 (2)
Br1—C1—C6—O1	−2.8 (2)	C4—C7—C12—C11	−175.62 (13)
Br1—C1—C6—C5	176.93 (12)	C8—C7—C12—C11	1.4 (2)
C2—C1—C6—O1	177.95 (14)	O3—C8—C9—C10	177.94 (14)
C2—C1—C6—C5	−2.4 (2)	O3—C8—C9—C15	−1.7 (2)
C1—C2—C3—O2	−178.78 (14)	C7—C8—C9—C10	0.5 (2)
C1—C2—C3—C4	1.5 (2)	C7—C8—C9—C15	−179.13 (13)
O2—C3—C4—C5	177.70 (14)	C8—C9—C10—C11	1.6 (2)
O2—C3—C4—C7	−5.9 (2)	C8—C9—C10—C16	−178.84 (14)
C2—C3—C4—C5	−2.6 (2)	C15—C9—C10—C11	−178.81 (14)
C2—C3—C4—C7	173.88 (14)	C15—C9—C10—C16	0.8 (2)
C3—C4—C5—C6	1.2 (2)	C9—C10—C11—O4	177.38 (14)
C7—C4—C5—C6	−175.45 (14)	C9—C10—C11—C12	−2.1 (2)
C3—C4—C7—C8	57.1 (2)	C16—C10—C11—O4	−2.2 (2)
C3—C4—C7—C12	−126.09 (16)	C16—C10—C11—C12	178.27 (14)
C5—C4—C7—C8	−126.50 (16)	O4—C11—C12—C7	−178.82 (14)
C5—C4—C7—C12	50.37 (19)	C10—C11—C12—C7	0.7 (2)

### Hydrogen-bond geometry (Å, º)

**Table:**

*D*—H···*A*	*D*—H	H···*A*	*D*···*A*	*D*—H···*A*
O3—H3*A*···O2	0.72 (3)	2.11 (3)	2.7576 (18)	152 (3)
O4—H4*A*···N1^i^	0.79 (2)	2.03 (2)	2.8189 (18)	172 (2)
C2—H2*A*···N2^ii^	0.93	2.72	3.638 (2)	168

Symmetry codes: (i) *x*+1, *y*, *z*; (ii) −*x*−1, *y*−1/2, −*z*−1/2.
